# New disease and old threats: A case series of COVID‐19 and tuberculosis coinfection in Saudi Arabia

**DOI:** 10.1002/ccr3.4233

**Published:** 2021-05-24

**Authors:** Mohammed Shabrawishi, Abdullmoin AlQarni, Maher Ghazawi, Baraa Melibari, Tebra Baljoon, Hassan Alwafi, Mohammed Samannodi

**Affiliations:** ^1^ Department of Medicine Al Noor Specialist Hospital Makkah Saudi Arabia; ^2^ Department of Radiology Al Noor Specialist Hospital Makkah Saudi Arabia; ^3^ Faculty of Medicine Umm Al‐Qura University Makkah Saudi Arabia; ^4^ Department of Medicine College of Medicine Umm Al‐Qura University Makkah Saudi Arabia

**Keywords:** COVID‐19, Co‐infection, Prognosis, Tuberculosis

## Abstract

COVID‐19 and TB coinfection are not common and may occur more in TB endemic countries. However, patients with pre‐COVID‐19 chronic respiratory symptoms should be screened for TB as well.

## Graphical abstract

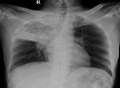

## 1 BACKGROUND

We present seven cases of COVID‐19 and TB coinfection. Our cases showed signs of TB before confirming the coinfection by microbiological tests. The prognosis was good, except for one patient who died. We conclude that COVID‐19 and TB coinfection are not common and may occur more in TB endemic countries.

COVID‐19, which started in China in December 2019, spreads to the world in the blink of an eye and became a global pandemic in March 2020. Since then (as of April 2020), there have been 133,146,550 confirmed cases and 2,88,530 deaths reported worldwide.^1^ Tuberculosis (TB) causes more deaths than any other infectious disease and the rate of latent infection is 25% of the global population.^2^ A recent study reported that patients with active or latent TB are more susceptible to COVID‐19 infection and severe forms of the disease.^3^ During the ongoing COVID‐19 pandemic, TB diagnosis may be missed or delayed due to similar clinical presentation. Therefore, clinicians should be cautious and consider the presence of TB when dealing with COVID‐19 patients, especially that both diseases may present with atypical presentations especially those in elderly.^4^, ^5^ To our knowledge, there have been only a few reports of COVID‐19 and TB coinfection in the literature.^6^, ^7^, ^8^, ^9^ Herein, we are reporting the first case series of seven cases of COVID‐19 and TB coinfection in Saudi Arabia.

## 2 CASE SERIES

### 2.1 Patient 1

**FIGURE 1:**
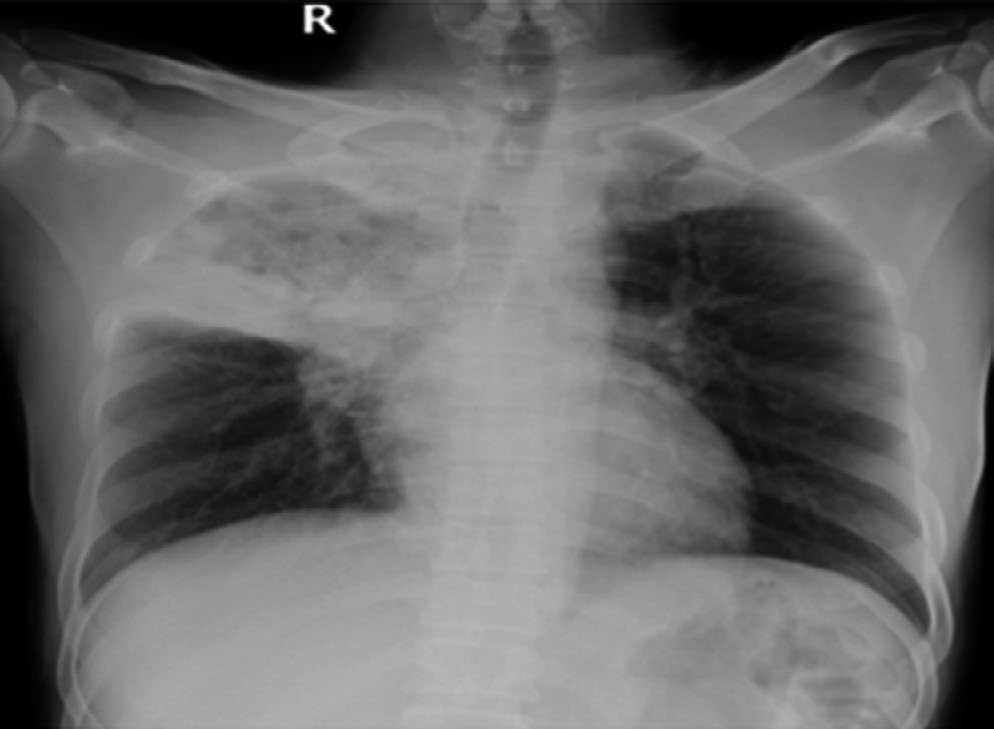
Frontal chest X‐ray showing lobar consolidation of the right upper lobe with central cavitation

A 42‐year‐old man, not known to have any underlying medical conditions, was admitted to the hospital as a suspected COVID‐19 case. He had complained of fever and cough for three months that had worsened three days prior to presentation. Fever was associated with generalized body aches and a productive cough with bloody sputum. Furthermore, there was a significant positive history of loss of appetite and loss of weight. There was no history of night sweats, shortness of breath (SOB), vomiting or diarrhea, and other systemic reviews were unremarkable. He denied any recent contact with a patient with TB. On admission, his vital signs were within normal limits. A physical examination revealed significant right‐sided decreased air entry with crepitation and no palpable lymph nodes. The blood workup for a complete blood count (CBC) and baseline chemistry panel was within the normal range, and the hepatitis B and C serologies and HIV workup were negative. The nasopharyngeal SARS‐CoV‐2 PCR (NSP) was positive, and a chest X‐ray showed right lobular consolidation and cavitation (Figure 1).

The coexistence of pulmonary TB was suspected due to the chronicity of symptoms, along with the suggestive radiological findings. Sputum for acid‐fast bacilli (AFB) and mycobacterium TB‐PCR (MTB) was sent. Later, the MTB PCR and the MTB culture were positive and a 4‐drug regimen of anti‐TB Rifampicin, Isoniazid, Pyrazinamide, and Ethambutol (RIPE) was initiated. Upon follow‐up, the patient became afebrile on the 4th day of hospitalization and was discharged to complete anti‐TB medications after repeated NSPs were negative on the 6th and 10th day of hospitalization. No significant adverse effect was noticed from the medication.

### 2.2 Patient 2

**FIGURE 2:**
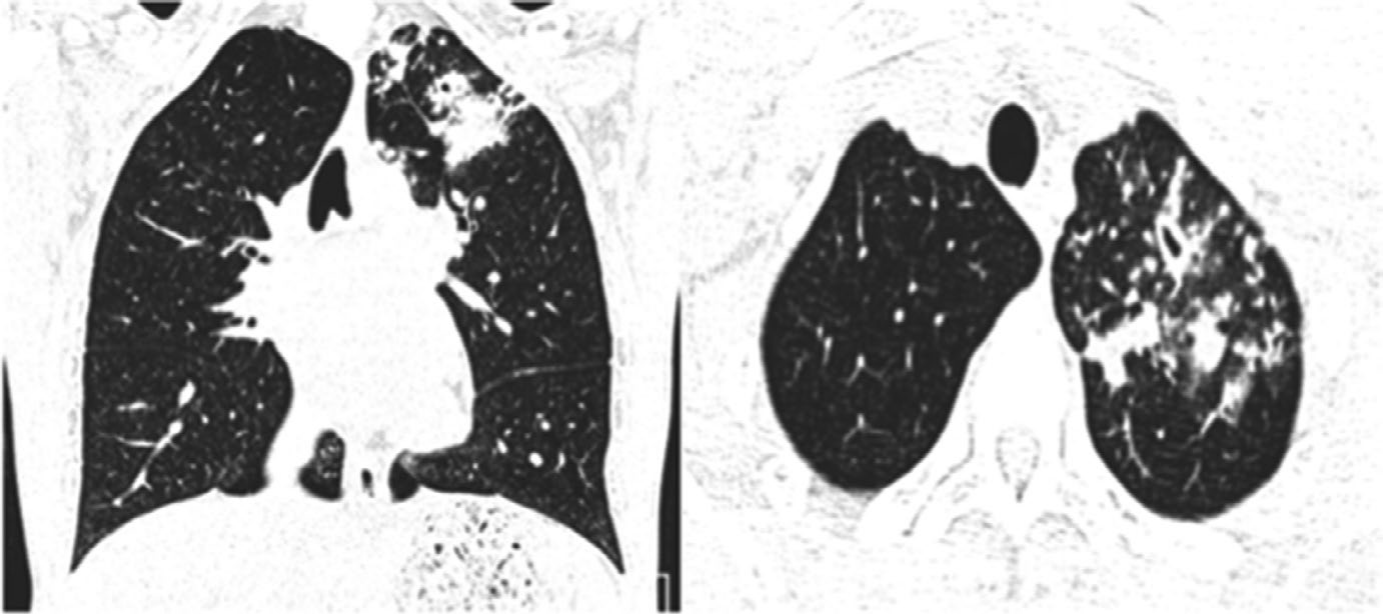
Atypical CT imaging features for COVID‐19. Axial (A) and coronal (B) CT images showing unifocal patchy consolidation with tree‐in‐bud opacities and centrilobular nodules

A 34‐year‐old man, free of underlying medical conditions, presented to the emergency department with a history of fever and cough for one month and a worsening cough for the two days prior to presentation. Fever was associated with significant subjective weight loss, night sweats, and loss of appetite. He denied a history of SOB, vomiting, or diarrhea. The rest of the systemic review was unremarkable. The patient had a significant history of close contact with a TB patient a few months prior to the presentation. The patient's vitals were within normal ranges except for a temperature of 38 degrees Celsius. The physical examination was normal as well, except for a palpable right axillary lymph node. Initial investigations were obtained, including CBC, chemistry, hepatitis serologies, and an HIV workup, which were unremarkable. Septic screening, a TB workup and an NSP sample, was sent. A chest X‐ray showed left upper lobe infiltration. Initially, the patient received ceftriaxone and azithromycin. Eventually, the NSP and MTB PCR were positive, while the AFB smear was negative. For further assessment, high‐resolution computed tomography (HRCT) of the lung was performed and showed unilateral left upper lobe patchy consolidation, centrilobular nodules, and tree‐in‐bud (Figure 2). He underwent a right axillary lymph node excisional biopsy that showed chronic granulomatous caseating inflammation. Thus, the patient was started on the anti‐TB regimen, RIPE. At follow‐up, the patient became afebrile only two days after starting anti‐TB treatment and had two negative swabs for COVID‐19 on the 4th and 7th day of admission. He was discharged with an improved clinical condition.

### 2.3 Patient 3

**FIGURE 3:**
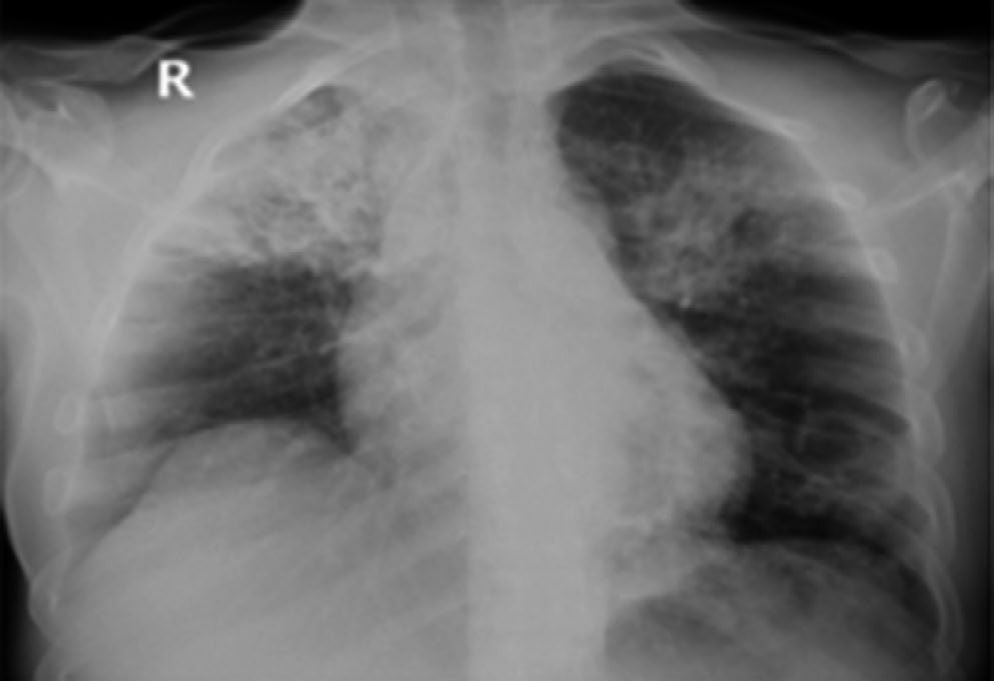
Frontal chest X‐ray showing lobar consolidation of the right upper lobe and sublobar consolidation of the left upper lobe with possible central cavitation

A 44‐year‐old man was hospitalized as a case of confirmed COVID‐19 for management. He complained of fever and cough for five days, associated with hemoptysis. Additionally, there was a positive history of night sweats and loss of appetite for one month. Upon admission, his temperature was 38.8 C. Physical examination revealed bilateral decreased breath sounds with fine crepitation. CBC and chemistry were within normal ranges. Initial ESR was 95 mm/h, and there was a CRP of 7.45 mg/dl. A chest X‐ray showed right lobar consolidation and cavitation (Figure 3). TB was considered, and sputum for acid‐fast bacilli (AFB), culture, and an MTB PCR were sent. Serology was negative for HIV and hepatitis B and C. The patient received azithromycin and hydroxychloroquine for a total of seven days. Subsequently, sputum AFB smears were positive, along with the MTB PCR; therefore, the patient was started on anti‐TB drugs (RIPE). At follow‐up, the patient had two consecutive negative NSPs on the 17th and 18th day of hospitalization. The patient was discharged after significant clinical improvement and two negative samples of sputum AFB smears. Later on, the culture grew a pan‐sensitive mycobacterium TB.

### 2.4 Patient 4

**FIGURE 4:**
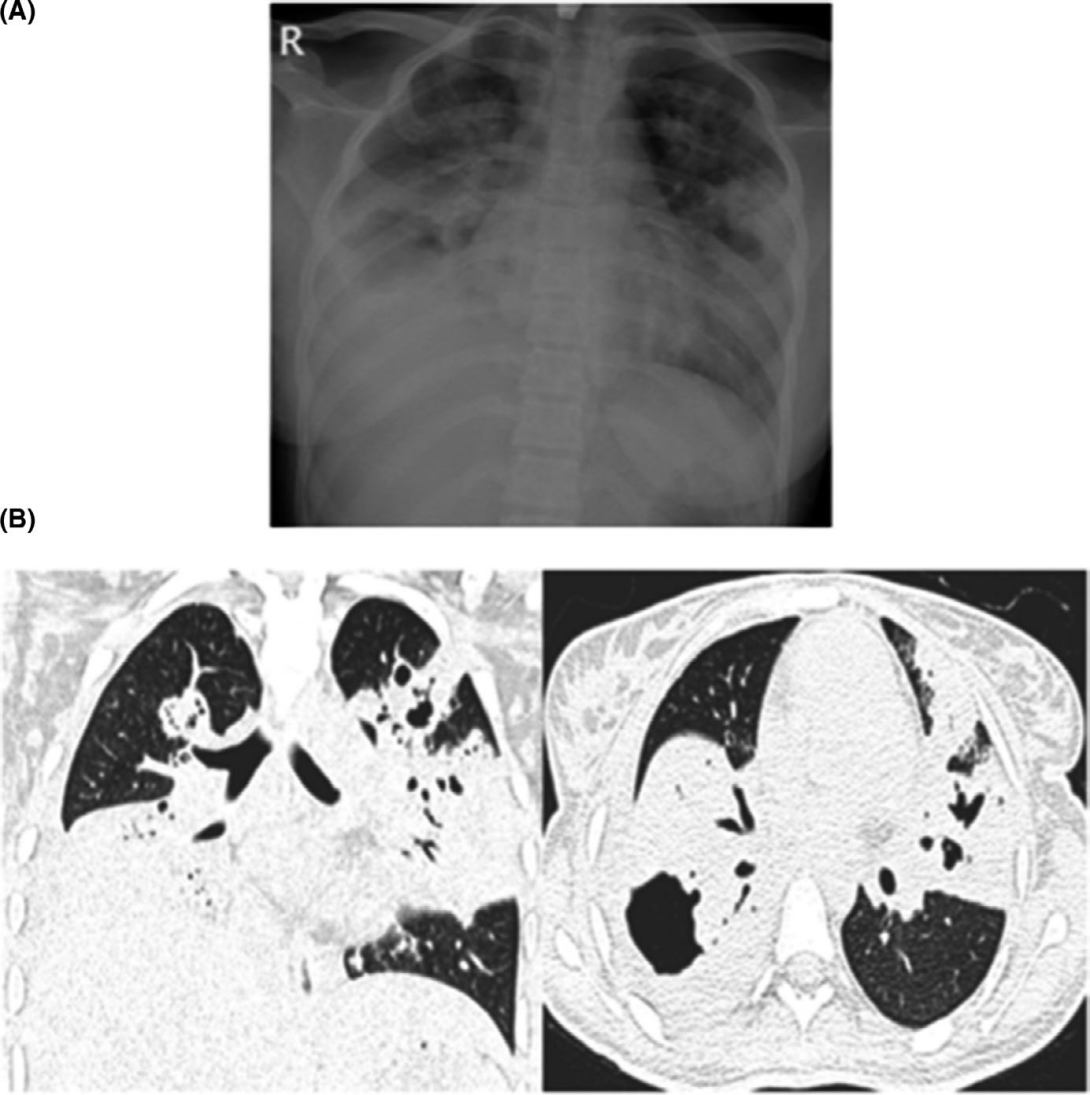
A, CXR showed bilateral patchy consolidation. B: CT chest images showing bilateral patchy consolidation, multifocal broncheactasis, unilateral pleural effusion, and calcified mediastinal lymphadenopathy

A 14‐year‐old girl was admitted to the hospital as a case of confirmed COVID‐19 for further management. She presented with a history of fever and dry cough for two weeks. The symptoms were associated with significant weight loss, night sweats, and loss of appetite. There was no prior history of pulmonary disease, but she reported contact with her roommate who tested positive for COVID‐19. At the time of presentation, her vitals were within normal ranges. A physical examination revealed decreased breath sounds, mainly on the right side, and no palpable lymph nodes were noticed. Routine blood tests revealed the following: hemoglobin levels of 9.4 g/dL, a WBC of 12 cells/mm, and a platelet count of 590.000/mm. Chemistry panels were within normal ranges, and ESR was 120 mm/hr and CRP 17mg/dl. A chest X‐ray revealed bilateral consolidation (Figure 4). The patient received ceftriaxone, azithromycin, and hydroxychloroquine. Coexistent pulmonary TB was considered, and samples for AFB smears and cultures were sent. HIV and hepatitis serologies were negative. The patient underwent an HRCT that showed bilateral patchy consolidation, bronchiectasis and cavitation, plus calcified hilar and mediastinal lymph node and unilateral pleural effusion (Figure 4). The sputum AFB smear was positive, and the cultures grew a pan‐sensitive MTB. Therefore, the patient was started on anti‐tubercular drugs (RIPE). At follow‐up, the patient became afebrile two days after starting the anti‐TB treatment and had two negative NSP swabs for COVID‐19 on the 5th and 8th day of admission repeatedly. The patient was discharged one month later after two negative AFB smears along with clinical improvement.

### 2.5 Patient 5

**FIGURE 5:**
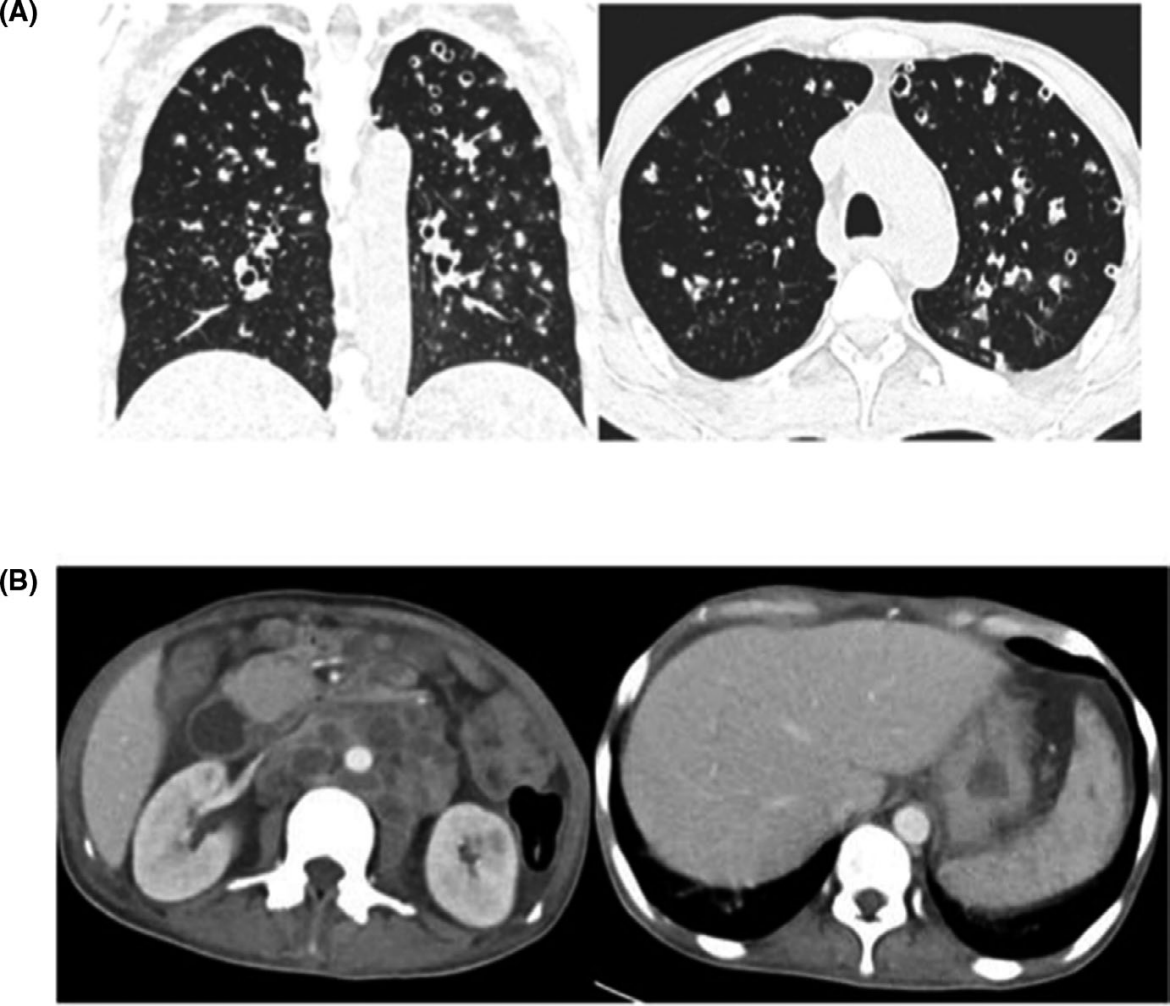
A: Atypical CT imaging features for COVID‐19. Unenhanced axial and coronal CT images showing diffuse tree‐in‐bud nodules with central cavitation. B, Contrast‐enhanced CT images of the abdomen and pelvis showing multiple necrotic lymph nodes with multifocal hypodense lesions in the spleen

A 30‐year‐old woman, not known to have any previous medical illness, presented to the emergency department with a complaint of intermittent fever for two weeks. The patient had a significant history of subjective weight loss, loss of appetite, and night sweats for the preceding couple of months. He denied any history of contact with sick patients, alcohol use, or illicit drug use. Upon presentation, the patient's temperature was 38.7 C, and he had a pulse rate of 110 beats per minute and blood pressure of 100/70 mm Hg. A physical examination revealed a significant cachectic ill appearance, decreased bilateral breath sounds, and no palpable lymph nodes. An initial investigation revealed a hemoglobin level of 6.8 g/dL, an MCV of 68, a WBC of 5 cells/mm, and a platelet count of 520 000/mm. The chemistry panel was within normal range. Hepatitis B and C serologies were negative; however, the HIV serology and the confirmatory test were positive, along with a CD4 count of less than 100. Initial ESR and CRP were >120 mm/hr and 18 mg/dL, respectively. A chest X‐ray revealed bilateral nodular opacities and patchy consolidation. Due to suggestive clinical presentation, a TB workup was sent and two consecutive sputum AFB smear samples were positive for AFB along with a positive MTB PCR. Furthermore, the NSP was positive. Subsequently, on follow‐up, the patient became leukopenic, with a WBC of 1.7 cells/mm. He underwent HRCT, which revealed patchy consolidation and ground‐glass opacification, cavitary nodules, plus tree‐in‐bud and necrotic axillary lymph nodes (Figure 5A). An abdominal CT showed significant necrotic upper abdominal lymph node, liver lesions, splenic lesions, and ascites suggestive of extrapulmonary TB (Figure 5B). The patient was treated with anti‐TB medications (RIPE) and triomethoprim‐sulfamethoxazole (TMP‐SMX). Follow‐up inflammatory markers revealed an ESR of 66 then 32mm/hr and a CRP of 11 mg/dl. Unfortunately, anti‐HIV medications could not be given due to the increased risk of immune reconstitution inflammatory syndrome (IRIS). Sadly, and due to extensive disease, the patient passed away.

### 2.6 Patient 6

**FIGURE 6:**
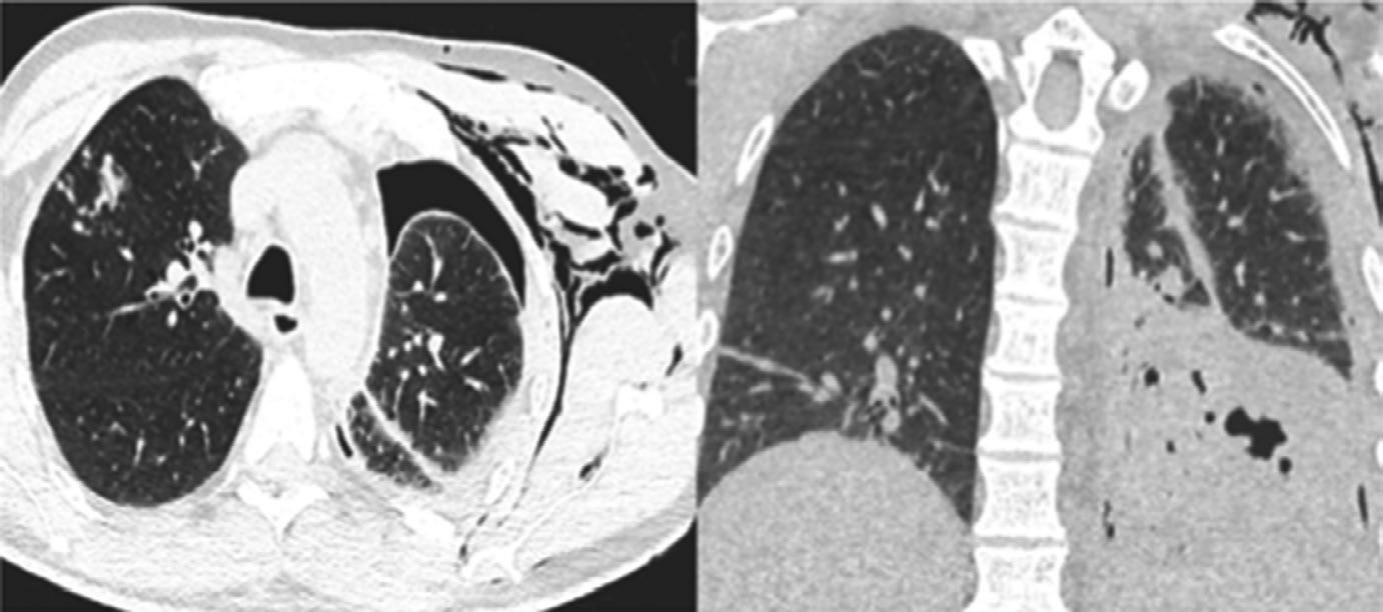
Atypical CT imaging features for COVID‐19. Unenhanced axial and coronal CT images showing tree‐in‐bud nodules with lobar consolidation of the left lower lobe and left side pneumothorax and subcutaneous emphysema

A 48‐year‐old man was referred to our hospital as a case of confirmed COVID‐19. He complained of intermittent fever and a dry cough for five days. On admission, the patient's temperature was 38.4 C, his pulse rate was 90 beats per minute, his respiratory rate was 20 breaths per minute, and his blood pressure was 140/85 mm Hg. A physical examination revealed decreased bilateral breath sounds and no palpable lymph nodes. Routine blood tests revealed the following: a hemoglobin level of 12 g/dL, a WBC of 14 cells/mm, a platelet count of 366.000/mm, and AST: 156 IU/L, ALT: 132 IU/L. A chest X‐ray revealed patchy consolidation with left side pneumothorax. Subsequently, the patient underwent chest CT, which revealed lobar consolidation, emphysema, plus tree‐in‐bud, hilar, and mediastinal lymph nodes (Figure 6). Blood and urine cultures were negative. Workup was negative for HIV and hepatitis B and C. Two samples of MTB PCR were positive, while AFB smears were negative. The patient was treated with triple antiviral therapy for COVID‐19, which includes ribavirin, interferon, and lopinavir/ritonavir, in addition to anti‐TB drugs (RIPE). At follow‐up, he was afebrile after seven days of admission and the first and second negative NSP were on the 9th and 15th day of admission. He was discharged after significant clinical improvement.

### 2.7 Patient 7

**FIGURE 7:**
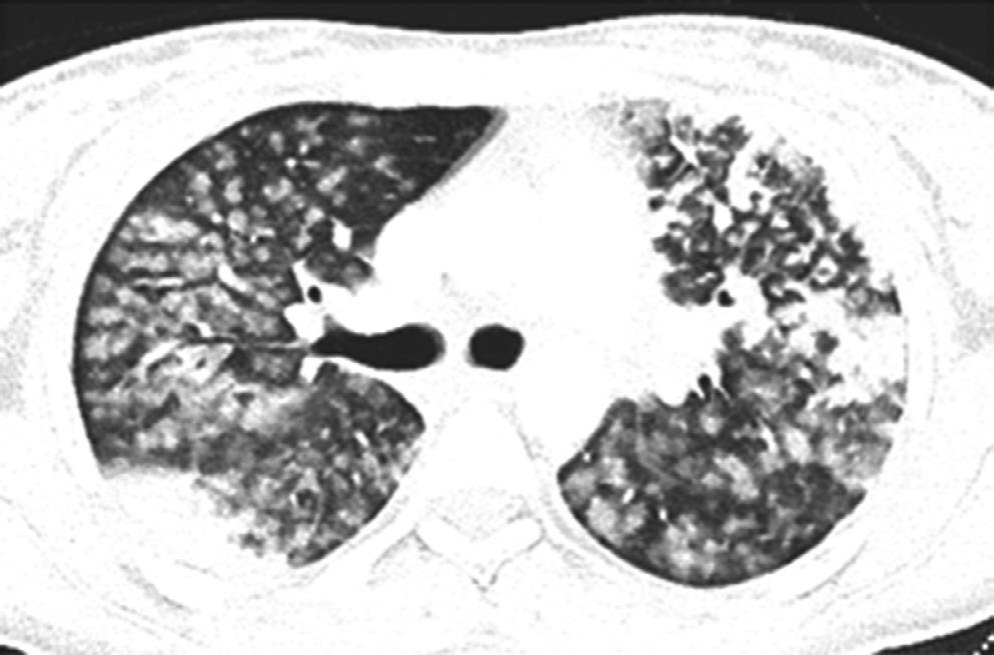
Atypical CT imaging features for COVID‐19. Unenhanced axial and coronal CT images showing diffuse centrilobular nodules with nonrounded peripheral consolidations

A 32‐year‐old diabetic woman presented to the emergency department complaining of having had a fever, dry cough, and dyspnea for five days. There was a history of weight loss and a loss of appetite for the previous two weeks. Upon admission, her physical examination revealed an ill‐looking lady with the following vital signs: an oxygen saturation in room air of 89%, a temperature of 38.8 C, blood pressure of 110/70 mmHg, a pulse rate of 120 beats per minute, and a respiratory rate of 27 breaths per minute. A chest examination revealed decreased breath sounds on the left side. There were no palpable lymph nodes. CBC and chemistry panels were within the normal ranges. A chest X‐ray showed nodular opacities and patchy consolidations bilaterally. The patient was admitted as a case of suspected COVID‐19. She received azithromycin and ceftriaxone. The patient subsequently tested positive for COVID‐19. Coexistent pulmonary TB was suspected due to clinical features and radiological findings. A sputum MTB PCR was positive, while AFB smears were negative. HIV and hepatitis serologies were negative. The initial CRP was 12mg/dl, and the ESR was 68 mm/hr At follow‐up, the patient's oxygen requirements progressively deteriorated and required further evaluation, with chest and abdominal CTs showing diffuse centrilobular nodules with nonrounded peripheral consolidations (Figure 7). The patient was treated with broad‐spectrum antibiotics and anti‐TB medication.

The patient was afebrile after five days, and her clinical symptoms had improved. The results of two samples of COVID‐19 were negative on the 4th and 11th day of admission. The patient was discharged on antitubercular medications and followed up at the pulmonology clinic.

## 3 DISCUSSION

We are reporting in our case series a rare and interesting coincidental finding of pulmonary TB and COVID‐19 in seven patients who had different presentations. Notably, both diseases mainly affect the respiratory systems; hence, it is important not to miss the possibility of coexistence of both diseases. Since the first few reported cases of COVID‐19 occurred, many reports had described the clinical characteristics of this disease.^10^, ^11^ The most commonly reported symptoms of COVID‐19 include cough, SOB, fever, and fatigue that mostly occur after a short incubation period.^12^ Less common symptoms include loss of smell and taste, sore throat, or gastrointestinal symptoms.^11^ In addition to the former symptoms, our cases and previously published cases of coinfection with TB experienced additional complaints such as hemoptysis,^9^, ^13^ night sweats,^8^ weight loss,^7^, ^9^ or loss of appetite. Hence, assessment for the presence of risk factors for TB infection such as close contact with a TB patient or being immigrants is helpful in determining coexistence. Furthermore, the suspicious radiological findings on presentation alerted the physicians for additional etiology other than COVID‐19 alone. Therefore, most of our patients, along with reported cases of coinfection of TB and COVID‐19,^6^, ^7^, ^8^, ^9^, ^13^, ^14^ had chronic symptoms and hence were likely to have undiagnosed former TB and subsequent COVID‐19 that led them to present to hospitals, rather than having TB deactivation secondary to COVID‐19.^15^

It is difficult to differentiate from the laboratory investigations between the two etiologies since CBC is nonspecific and a WBC count can be normal, elevated, or reduced; however, the finding of anemia can suggest chronic disease. Elevation of the liver enzymes and inflammatory markers can occur in both diseases. However, the severity and duration of improvement can be of value as it takes a longer time for TB patients to return to normal values. Specific testing for TB through samples from the respiratory tract should be considered when these, along with the presentation mentioned earlier and the radiological findings, are not typical for COVID‐19. In our study, all patients had pan‐sensitive MTB and this also suggests that coexistence does not affect sensitivity. Additional extrapulmonary TB was found in one patient that was found to have HIV.

The presence of indeterminate or atypical radiological features for COVID‐19 specifically should raise the possibility of an incidental alternative disease. Our cases highlight the importance of analyzing such radiological findings to suggest an alternative diagnosis or a potential coinfection with another viral or bacterial disease (like TB). The Radiological Society of North America proposed four categories for the suggested standardized CT reporting language of COVID‐19 based on current literature and expert consensus.^16^

Apart from assessing association, the other main concern about coinfection of COVID‐19 and TB is poorer treatment outcomes and may also lead to underdiagnosis of TB,^17^ especially if TB treatment is interrupted.^2^ Pre‐existing TB and underlying lung disease will affect the clinical severity of COVID‐19. Furthermore, TB itself or the use of immunomodulators (IL‐6 inhibitors) in moderate‐severe COVID‐19 may lead to reactivation of latent TB in high endemic areas.^18^ The other association is the possibility of drug‐drug interactions (eg, rifampicin and lopinavir/ritonavir) and additive hepatotoxicity (remdisivir) due to simultaneous use of antitubercular drugs and available COVID‐19 therapeutic options.

In general, the outcome of COVID‐19 is variable. In the largest report, which was published from Wuhan, China, the mortality rate was less than 2%. The mortality was higher in the elderly population with comorbidities such as diabetes, hypertension, ischemic heart disease, and chronic obstructive pulmonary disease.^12^ Later, Zhou et al added more risk factors for death, including d‐dimer levels greater than 1 μg/mL, and higher SOFA scores on admission.^19^ Interestingly, the mortality rate increased significantly with COVID‐19 and pulmonary TB co‐infection. Tadolini et al have a reported mortality rate of 10.2% with COVID‐19 and pulmonary TB coinfection, which was higher in elderly patients with multiple comorbidities.^6^ Similarly, coinfection led to more deaths, according to the two cohorts of Motta et al (14.3% and 10.2%, respectively).^20^

In our study, the majority of patients had a good prognosis with full recovery except for one patient who died. The estimated mortality rate was 14.3%. We believe that the severity of the underlying AIDS condition with disseminated TB and COVID‐19 were the main factors that led to death in this patient.

The COVID‐19 pandemic had a huge negative impact on many healthcare‐related issues and essential TB control, such as the delivery of diagnostic tools, prophylactics, and mediation regimens. Hence, this could lead to terrifying scenarios such as decreased adherence to medications, giving rise to increasing numbers of drug‐resistant cases, and a decrease in the quality of care could also increase deaths from TB that occur annually worldwide. As mentioned earlier, similar clinical presentations of COVID‐19 and TB might result in patients hesitating to seek medical care due to fear of COVID‐19, although the symptoms may result from TB, leading to worsening outcomes. Due to overwhelming demand or restrictions applied in many healthcare facilities, there are subtle presentations that are likely to be missed. The survival of this vulnerable group depends on the time of diagnosis, which will render the situation more challenging. On the other hand, this situation may cause delayed diagnosis of newly infected TB patients, difficulties in accessing medications, and impaired TB treatment monitoring. Moreover, this situation can result in increased coronavirus transmission for those who live in hustles and among homeless individuals.

Similar consideration needs to be given to COVID‐19/HIV coinfection patients as well, especially when there are not enough data currently to inform us of the sequelae of both coinfections. One positive thing learned from the COVID‐19 pandemic is that prevention is better than cure in TB too, and that TB needs more attention than ever. Taken together, to protect the individual from additional risks, screening for COVID‐19 symptoms should contain questions about TB as well.

## CONFLICT OF INTEREST

The authors declare no conflict of interest.

## AUTHOR CONTRIBUTIONS

MSh and AA: contributed to conception and design of the study, acquisition and analysis of data, and drafting a significant portion of the manuscript. MG and TB: contributed to conception and design of the study, and acquisition and analysis of data. BM: contributed to acquisition and analysis of data. MS: contributed to conception and design of the study, acquisition and analysis of data, and drafting a significant portion of the manuscript. All authors agreed to be accountable for the content of the work.

## INFORMED CONSENT

Informed consent was obtained.

## Data Availability

No further data are available.
